# Supplementary material for: Functional consequences of the long-term decline of reef-building corals in the Caribbean: evidence of across-reef functional convergence
Source: R Soc Open Sci. 2019 Oct 23;6(10):190298. doi: 10.1098/rsos.190298 (PMC6837220; doi:10.1098/rsos.190298)
Supplement: Figure S1 and Tables S1 - S4 [file rsos190298supp1.docx]

**Supplementary information**

Table S1. Classification of the coral species recorded in this study according to four morpho-functional groups (see methods) and colony morphology (obtained from Reyes-Bonilla & Jordan-Dahlgren (2017)^1^.

| **Coral species** | **Morpho-functional group** | **Colony morphology** |
| --- | --- | --- |
| ***Agaricia agaricites*** | Non-framework | Sub-massive or encrusting |
| ***Acropora cervicornis*** | Framework-building branching | Branching |
| ***Agaricia grahamae*** | Non-framework | Foliose |
| ***Acropora palmata*** | Framework-building branching | Branching |
| ***Acropora prolifera*** | Framework-building branching | Branching |
| ***Agaricia humilis*** | Non-framework | Sub-massive or encrusting |
| ***Agaricia lamarcki*** | Non-framework | Foliose |
| ***Agaricia tenuifolia*** | Foliose-digitiform | Foliose |
| ***Colpophyllia natans*** | Massive | Massive |
| ***Dendrogyra cylindrus*** | Massive | Massive |
| ***Diploria labyrinthiformis*** | Massive | Massive |
| ***Dichocoenia stokesii*** | Non-framework | Small massive |
| ***Eusmilia fastigiata*** | Non-framework | Small branching |
| ***Favia fragum*** | Non-framework | Small massive |
| ***Helioseris cucullata*** | Non-framework | Foliose/platty |
| ***Isophyllia rigida*** | Non-framework | Small massive |
| ***Isophyllia sinuosa*** | Non-framework | Small massive |
| ***Madracis aurentenra*** | Non-framework | Digitiform |
| ***Madracis decactis*** | Non-framework | Digitiform |
| ***Meandrina meandrites*** | Massive | Massive |
| ***Manicia areolata*** | Non-framework | Sub-massive |
| ***Montastraea cavernosa*** | Massive | Massive |
| ***Mycetophyllia lamarckiana*** | Non-framework | Foliose |
| ***Mussa angulosa*** | Non-framework | Small massive |
| ***Orbicella annularis*** | Massive | Massive |
| ***Orbicella faveolata*** | Massive | Massive |
| ***Orbicella franksi*** | Massive | Massive |
| ***Porites astreoides*** | Non-framework | Small massive |
| ***Pseudodiploria clivosa*** | Massive | Massive |
| ***Porites divaricata*** | Non-framework | Digitiform |
| ***Porites furcata*** | Non-framework | Digitiform |
| ***Porites porites*** | Foliose-digitiform | Digitiform |
| ***Pseudodiploria strigosa*** | Massive | Massive |
| ***Solenastrea bournoni*** | Non-framework | Small massive |
| ***Stephanocoenia intersepta*** | Non-framework | Sub-massive or encrusting |
| ***Siderastrea radians*** | Non-framework | Sub-massive or encrusting |
| ***Siderastrea siderea*** | Massive | Massive |
| ^1^Reyes-Bonilla, H., & Jordán-Dahlgren, E. (2017). Caribbean Coral Reefs: Past, Present, and Insights into the Future. *Marine Animal Forests: The Ecology of Benthic Biodiversity Hotspots*, 31-72. | | |

**Table S2.** Mann-Whitney U-test values

| Years | Reef zone | Variable | U-value | P-value | Confidence interval |
| --- | --- | --- | --- | --- | --- |
| 1985 vs. 2016 | Back-reef | Coral cover | 334 | 0.004 | y1=-19.833291 y2=-3.333358 |
| 1985 vs. 2016 | Fore-reef | Coral cover | 200 | 0.3402 | y1= -3.499965 y2= 11.833350 |
| 1985 vs. 2016 | Back-reef | Framework-building branching | 847.5 | 0.0000026 | y1=1.529743e-05 y2=4.499947e+00 |
| 1985 vs. 2016 | Back-reef | Foliose-digitiform | 799 | 0.00005067 | y1=7.190187e-05 y2=2.000051e+00 |
| 1985 vs. 2016 | Back-reef | Massive | 452.5 | 0.1627 | y1=-7.500005 y2=0.833340 |
| 1985 vs. 2016 | Back-reef | Non-framework | 600 | 0.2399 | y1=-7.500005 y2=0.833340 |
| 1985 vs. 2016 | Fore-reef | Framework-building branching | 183 | 0.556 | y1= -8.784154e-06 y2= 3.985645e-05 |
| 1985 vs. 2016 | Fore-reef | Foliose-digitiform | 154 | 0.5837 | y1= -3.027599e-05 y2= 3.782326e-05 |
| 1985 vs. 2016 | Fore-reef | Massive | 156 | 0.7274 | y1= -6.500027 y2= 6.499931 |
| 1985 vs. 2016 | Fore-reef | Non-framework | 90 | 0.01857 | y1= -4.3333978 y2= -0.4999497 |


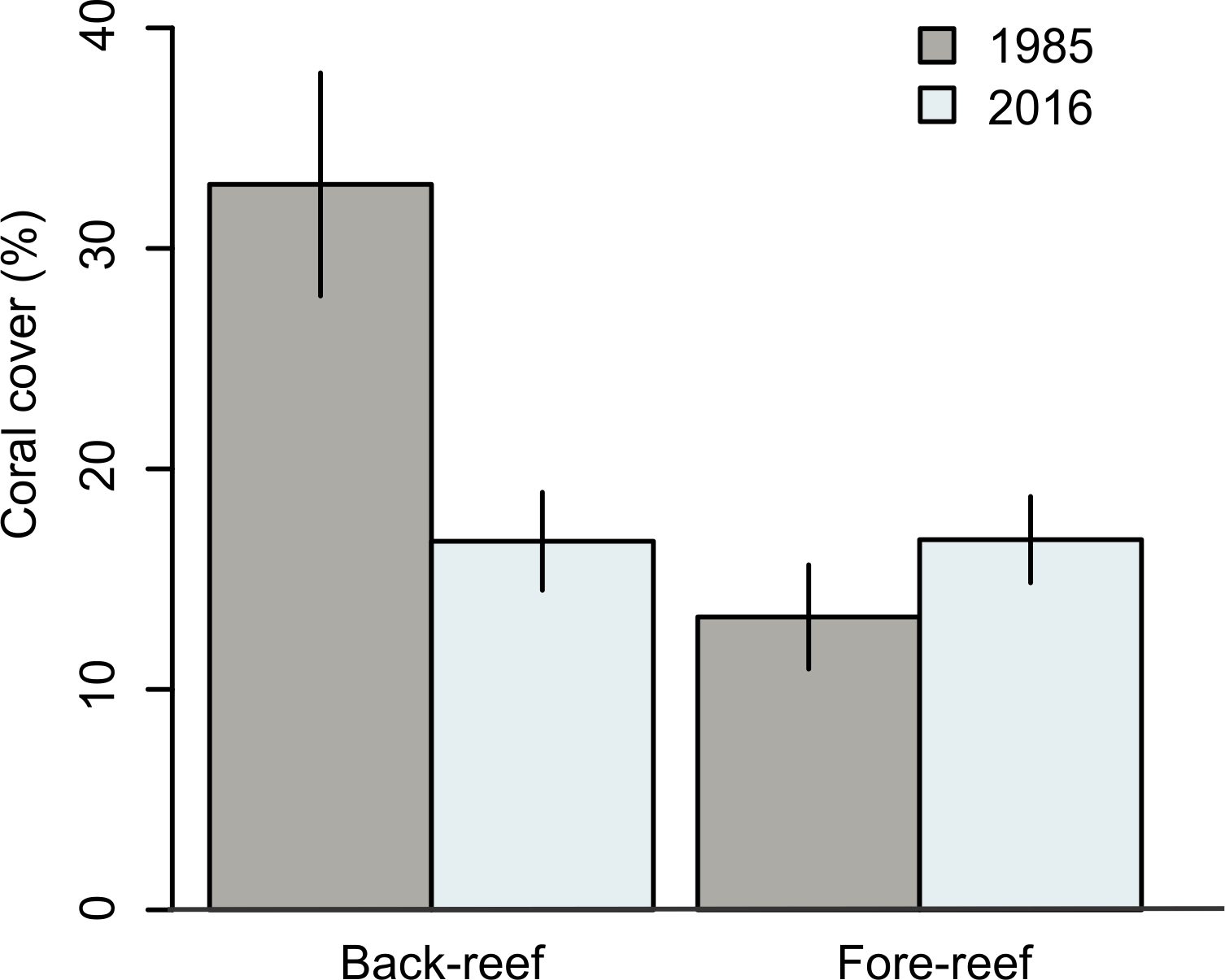


**Figure S1. Average coral cover for the studied sites for the back- and fore-reef zones.**  The error bars are the confidence intervals from the mean

Table S3. Coral cover (mean±SE) on the surveyed sites (n = 16) with their respective reef-zone for each year.

| Site | Year | Reef zone | Coral cover (%) |
| --- | --- | --- | --- |
| Bocana | 1985 | Back | 35.5 ±8.6 |
| Bonanza | 1985 | Back | 16.8±6.3 |
| Bonanza | 1985 | Fore | 4±1.1 |
| Maroma | 1985 | Fore | 28.5±2.4 |
| Nizuc | 1985 | Fore | 20.1±3.7 |
| Petempich | 1985 | Fore | 37.4±14.2 |
| Puerto Morelos | 1985 | Back | 33.8±4.5 |
| Bonanza Profundo | 2016 | Fore | 15.5±3.2 |
| Bonanza | 2016 | Back | 7.3±2.3 |
| La Bocana | 2016 | Back | 20.8±4.8 |
| MX1062 | 2016 | Fore | 5.7±1.7 |
| Punta Maroma Norte | 2016 | Fore | 24.7±3.6 |
| Punta Maroma Sur | 2016 | Fore | 21.3±2.9 |
| Radio Pirata | 2016 | Back | 20.8±3.5 |
| Tanchacté Norte | 2016 | Back | 16.6±4.1 |
| Tanchacté Sur | 2016 | Back | 18.6±3.1 |

**Table S4.** Mann-Whitney U-test values of the reef function parameters

| Years | Reef zone | Variable | W-value | P-value | Confidence interval |
| --- | --- | --- | --- | --- | --- |
| 1985 vs. 2016 | Back-reef | Coral carbonate production | 755 | 0.01909 | y1=0.1212755 y2=1.6372102 |
| 1985 vs. 2016 | Back-reef | Functional Reef Index | 795 | 0.004515 | y1= 0.02737289 y2= 0.18858781 |
| 1985 vs. 2016 | Back-reef | Net carbonate production | 755 | 0.01909 | y1=0.1212755 y2=1.6372102 |
| 1985 vs. 2016 | Fore-reef | Coral carbonate production | 124 | 0.3767 | y1=-0.8126667 y2=0.2479000 |
| 1985 vs. 2016 | Fore-reef | Functional Reef Index | 124 | 0.1906 | y1=-0.16145356 y2=0.02694995 |
| 1985 vs. 2016 | Fore-reef | Net carbonate production | 138 | 0. 3767 | y1=-0.8126667 y2=0.2479000 |

Table S5. Summary of bioerosion rates for each group of bioeroder and the carbonate production for each functional group.

| Bioerosion rates | | |
| --- | --- | --- |
| Zone | **Group** | **Mean bioerosion (G) ± SD** |
| Back-reef | Parrotfish | 3.32±0.33 |
| Back-reef | Microbioerosion | 0.25±0.06 |
| Back-reef | Macrobioerosion (sponges) | 0.004±0.002 |
| Back-reef | Urchins | 0.09±0.15 |
| Fore-reef | Parrotfish | 1.50±1.49 |
| Fore-reef | Microbioerosion | 0.25±0.07 |
| Fore-reef | Macrobioerosion (sponges) | 0.02±0.003 |
| Fore-reef | Urchins | 0±0 |

| Carbonate production | | | |
| --- | --- | --- | --- |
| Zone | **Year** | **Group** | **Mean production(G) ± SD** |
| Back-reef | 1985 | Branching | 4.91±5.98 |
| Back-reef | 1985 | Foliose digitiform | 0.23±0.38 |
| Back-reef | 1985 | Massive | 0.21±0.31 |
| Back-reef | 1985 | Non-framework | 0.10±0.13 |
| Fore-reef | 1985 | Branching | 0.58±0.45 |
| Fore-reef | 1985 | Foliose digitiform | 0.04±0.02 |
| Fore-reef | 1985 | Massive | 0.16±0.2 |
| Fore-reef | 1985 | Non-framework | 0.06±0.06 |
| Back-reef | 2016 | Branching | 0.28± |
| Back-reef | 2016 | Foliose digitiform | 0.11±0.20 |
| Back-reef | 2016 | Massive | 1.37±1.28 |
| Back-reef | 2016 | Non-framework | 0.17±0.18 |
| Fore-reef | 2016 | Branching | 0.41±0.23 |
| Fore-reef | 2016 | Foliose digitiform | 0.07±0.05 |
| Fore-reef | 2016 | Massive | 0.98±0.83 |
| Fore-reef | 2016 | Non-framework | 0.23±.01 |


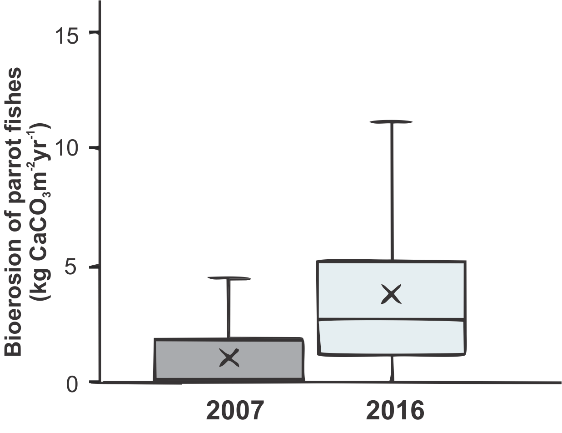


**Figure S2.** Box and whisker plot of bioerosion from parrotfishes for back-reef sites in 2007 and 2016. The bottom and top of the box are the first and third quartiles, respectively, the “x” is the average and the black line is the median. Whiskers are the minimum and maximum, the notches.
